# 2019 EULAR points to consider for non-physician health professionals to prevent and manage fragility fractures in adults 50 years or older

**DOI:** 10.1136/annrheumdis-2020-216931

**Published:** 2020-04-24

**Authors:** Jo Adams, Nicky Wilson, Emalie Hurkmans, Margot Bakkers, Petra Balážová, Mark Baxter, Anne-Birgitte Blavnsfeldt, Karine Briot, Catharina Chiari, Cyrus Cooper, Razvan Gabriel Dragoi, Gabriele Gäbler, Willem Lems, Erika Mosor, Sandra Pais, Cornelia Simon, Paul Studenic, Simon Tilley, Jenny de la Torre-Aboki, Tanja A Stamm

**Affiliations:** 1 School of Health Sciences, University of Southampton, Southampton, UK; 2 Department Care I, Musculoskeletal System & Neurology, Dutch National Health Care Institute, Diemen, The Netherlands; 3 EULAR Standing Committee of People with Arthritis/Rheumatism in Europe (PARE), Zurich, Switzerland; 4 EULAR Young PARE, Zurich, Switzerland; 5 Slovak League Against Rheumatism, Piestany, Slovakia; 6 Medicine for Older People, University Hospital Southampton NHS Foundation Trust, Southampton, UK; 7 Department of Rheumatology, Aarhus University Hospital, Arrhus, Denmark; 8 INSERM U1153, Paris Descartes University, Reference Center for Genetic Bone Diseases - Department of Rheumatology, Cochin Hospital, Paris, France; 9 Department of Orthopedics and Trauma-Surgery, Medical University of Vienna, Vienna, Austria; 10 MRC Lifecourse Epidemiology Unit, University of Southampton, Southampton, UK; 11 Rehabilitation, Physical Medicine and Rheumatology, 'Victor Babes' University of Medicine and Pharmacy, Timisoara, Timisoara, Romania; 12 Section for Outcomes Research, Centre for Medical Statistics, Informatics, and Intelligent Systems, Medical University of Vienna, Vienna, Austria; 13 Department of Rheumatology, VU University Medical Centre Amsterdam, Amsterdam, Noord-Holland, The Netherlands; 14 Centre for Biomedical Research, Department of Biomedical Sciences and Medicine, University of Algarve, Faro, Portugal; 15 Department of Balneology, Rehabilitation and Rheumatology, 'Victor Babes' University of Medicine and Pharmacy, Timisoara, Timisoara, Romania; 16 Internal Medicine 3, Division of Rheumatology, Medical University Vienna, Vienna, Austria; 17 Trauma & Orthopaedics, University Hospital Southampton NHS Foundation Trust, Southampton, UK; 18 Day Hospital, Alicante General and university Hospital, Alicante, Spain; 19 Ludwig Boltzmann Institute Arthritis and Rehabilitation, Vienna, Austria

**Keywords:** osteoporosis, multidisciplinary team-care, health services research

## Abstract

**Objective:**

To establish European League Against Rheumatism (EULAR) points to consider for non-physician health professionals to prevent and manage fragility fractures in adults 50 years or older.

**Methods:**

Points to consider were developed in accordance with EULAR standard operating procedures for EULAR-endorsed recommendations, led by an international multidisciplinary task force, including patient research partners and different health professionals from 10 European countries. Level of evidence and strength of recommendation were determined for each point to consider, and the mean level of agreement among the task force members was calculated.

**Results:**

Two overarching principles and seven points to consider were formulated based on scientific evidence and the expert opinion of the task force. The two overarching principles focus on shared decisions between patients and non-physician health professionals and involvement of different non-physician health professionals in prevention and management of fragility fractures. Four points to consider relate to prevention: identification of patients at risk of fracture, fall risk evaluation, multicomponent interventions to prevent primary fracture and discouragement of smoking and overuse of alcohol. The remaining three focus on management of fragility fractures: exercise and nutritional interventions, the organisation and coordination of multidisciplinary services for post-fracture models of care and adherence to anti-osteoporosis medicines. The mean level of agreement among the task force for the overarching principles and the points to consider ranged between 8.4 and 9.6.

**Conclusion:**

These first EULAR points to consider for non-physician health professionals to prevent and manage fragility fractures in adults 50 years or older serve to guide healthcare practice and education.

**Key messages**

**What is already known about this subject?**

- Interventions delivered by non-physician health professionals to prevent and manage fragility fractures contribute to optimal patient outcomes. They have not been sufficiently covered to date in existing European League Against Rheumatism/European Federation of National Associations of Orthopaedics and Traumatology recommendations.

**What does this study add?**

- This paper will guide clinical practice in Europe regarding interventions delivered by non-physician health professionals to prevent and manage fragility fractures in adults 50 years or older. Several areas described in this paper highlight the necessity for further research. Future studies could build on our findings. International and national initiatives may find our paper useful as a common European reference.
- Prevention of fragility fractures is essential for good health in older age; osteoporosis and fractures are key issues that need to be considered. Especially vulnerable patient groups, for example, frail older people, and those with cognitive impairments will benefit from European standards regarding interventions delivered by non-physician health professionals to prevent and manage osteoporotic fractures.
- Implementation will be supported by national organisations, professional and scientific societies, including patient leagues.

**Key messages**

**How might this impact on clinical practice or future developments?**

- Improved care delivered by non-physician health professionals to prevent and manage fragility fractures offers opportunities for better health outcomes in older people in Europe.

## Introduction

Countries across the world are facing a fragility fracture crisis.^1^ Estimates suggest that by 2040 over 300 million adults age 50 years or more worldwide will be at high-risk of fragility fracture.^2^ In 2017, across France, Germany, Italy, Spain, Sweden and the UK alone, there were 2.68 million new fragility fractures, costing an estimated €37.5 billion.^3^ These numbers are projected to rise, such that in 2030 over 3.3 million new fractures are anticipated across the same six countries, with accompanying total fracture-related costs approximating €47.4 billion.^3^

Many fragility fractures require immediate acute fracture care and typically lead to physical disability, persistent pain, impaired quality of life and increased mortality.^4^ Among those who sustain a fragility fracture, the risk of imminent subsequent fracture is substantial,^5 6^ highlighting the importance of primary and secondary fracture prevention.

Interventions delivered by non-physician health professionals (HPs), such as dietitians, nurses, occupational therapists, pharmacists and physiotherapists, in close collaboration with rheumatologists, orthopaedic surgeons, rehabilitation specialists and general practitioners, are important in the management of patients at high-risk of fragility fractures. Interventions by non-physician HPs include exercise and functional training, prescription of assistive devices, fall prevention programmes, nutritional supplements and education. Drug therapy is important in the prevention and management of fractures, and in some countries non-physician HPs can prescribe anti-osteoporosis medicines.^7^

The European League Against Rheumatism (EULAR) Standing Committees recognise the importance of optimising healthcare delivered by non-physician HPs to people at high-risk of fragility fractures. The EULAR/EFORT (European Federation of National Associations of Orthopaedics and Traumatology) recommendations for management of patients older than 50 years with a fragility fracture and prevention of subsequent fracture,^8^ focussed primarily on physician-based interventions. Interventions delivered by non-physician HPs were not comprehensively covered. Therefore, this study aimed to establish EULAR points to consider for the prevention and management of fragility fractures by non-physician HPs to complement and extend the EULAR/EFORT recommendations. As there is considerable variation across European countries in the roles and tasks of HPs, we focussed on interventions that could potentially be delivered by non-physician HPs independent of whether specific HPs do certain interventions in a country or not.

## Methods

Points to consider were developed in accordance with up-to-date EULAR standard operating procedures for EULAR-endorsed recommendations.^9^ An international multidisciplinary task force was established, comprising two patient research partners, one dietitian, one geriatrician and one nurse, three occupational therapists, two orthopaedic surgeons, four physiotherapists, one specialist in physical medicine and rehabilitation and five rheumatologists, with expertise in the management of osteoporosis and/or fragility fractures. A Delphi survey, conducted by email, was undertaken to set up and prioritise the clinical questions on a 9-point Likert-scale (scores 1 to 3 ‘not relevant’, scores 4 to 6 ‘potentially relevant’, scores 7 to 9 ‘(highly) relevant’). Thirteen questions were reduced to eight via two rounds of voting by the task force (questions scoring <4 were excluded, questions scoring >6 were included and questions scoring 4 to 6 were discussed and revised). This was followed by a systematic literature review (SLR) based on the eight clinical questions (online supplementary file 1, table 1) formulated around two linked concepts: (i) adults ≥50 years of age at high-risk of primary or secondary osteoporotic fracture and (ii) interventions delivered by non-physician HPs to prevent and manage osteoporotic fractures. High-risk of osteoporotic fracture was categorised based on bone mineral density (BMD) values for osteoporosis and osteopenia^10^ and/or short-term probability of fracture (table 1). Key outcomes were fractures and falls, although BMD and risk of falling were included as surrogate endpoints.

10.1136/annrheumdis-2020-216931.supp1 Supplementary data

**Table 1:** Categorisation of individuals at high-risk of fragility fracture

Osteopenia	T score =<-1.0 to −2.5 SD
Osteoporosis	T score =≤−2.5 SD
FRAX 10-year probability of a major* osteoporotic fracture	≥20% (age independent)
FRAX 10-year probability of hip fracture	≥3% (age independent)
FRAX NOGG threshold	40 to 90 years (age dependent)

Note: T score, unit of SD from the mean for bone mineral density compared with a healthy young adult; FRAX, Fracture Risk Assessment Tool; NOGG, National Osteoporosis Guideline Group.

FRAX intervention thresholds vary between countries.

*A clinical spine, hip, forearm or humerus fracture.

Evidence was appraised using a domain-based assessment of risk of bias for primary studies,^11^ and A MeaSurement Tool to Assess systematic Reviews (AMSTAR 2)^12^ and classified using the Oxford 2011 Levels of Evidence Table^13^ (online supplementary file 1, tables 2-5). Evidence was rated as: sufficient, some, insufficient and insufficient evidence to determine^14^ (online supplementary file 1, table 6). The research fellow (NW), and one convenor (EH), extracted data for the SLR in close collaboration with the methodologist (TAS). This SLR has been published.^15^

**Table 2:** Overarching principles and EULAR points to consider for the prevention and management of fragility fracture by non-physician HPs

**No**	**Overarching principles**			**Level of Agreement (Mean (SD))**
**1**	**The management of patients at risk of a fragility fracture should be based on shared decision making between patients and non-physician HPs**.			9 (1.8)
**2**	**Non-physician HPs should be involved in the management of patients at risk of fragility fractures**.			8.4 (2.2)
**No**	**Point to consider**	**Level of evidence**	**Strength of recommendation**	**Level of Agreement (Mean (SD))**
	**Prevention of Fragility Fractures**			**Median (Range)**
**1**	**Identification of patients at risk of fracture**			
	Non-physician HPs should identify patients at risk of fragility fracture, ensure that the patients are offered opportunities for adequate treatment and address bone fragility in patient education.			9.06 (1.16)
		2	B	
				9.5 (7–10)
**2**	**Fall risk evaluation**			
	Non-physician HPs should start with fall risk evaluation of patients at risk of fragility fracture. Patients at high-risk of falls should be assessed by non-physician HPs using an individualised approach to multi-component screening or referred to one or more non-physician HPs competent in multi-component screening.	4	C	9.61 (0.70)
				10 (8 to 10)
**3**	**Preventive multicomponent interventions**			
	Tailored multicomponent interventions, including for example:			
	Exercise	1 to 3	A	9.33 (0.91)
	Environmental adaptations	2	D	
	Nutrition	1 to 2	D	10 (8 to 10)
	Education	2	D	
	should be offered to patients at high-risk of primary osteoporotic fracture and/or high-risk of falls			
**4**	**Avoidance of smoking and overuse of alcohol**			
	Smoking and overuse of alcohol should be discouraged.	1	A	9.22 (1.31)
				10 (5 to 10)
**No**	**Point to consider**	**Level of evidence**	**Strength of recommendation**	**Level of Agreement (Mean (SD))**
	**Management of Fragility Fractures**			**Median (Range)**
**5**	**Exercise and nutritional interventions for patients who have experienced a fragility fracture**			
	Non-physician HPs should ensure that patients who have experienced a fragility fracture are given opportunities for:			
	adequate exercise	1 to 2	A	9.22 (0.88)
	adequate nutritional intake	2	D	
	Calcium and vitamin D intake should be discussed with the patient focussing on actual and recommended daily calcium intake, calcium and vitamin D rich foods, and the individual’s risk/benefit profile for vitamin D supplementation.	1 to 2	D	9.5 (8 to 10)
**6**	**Organisation and coordination of multidisciplinary services**			
	Non-physician HPs should be included in orthogeriatric services, FLS and/or a coordinated, multidisciplinary post-fracture prevention programme. Patients with fragility fractures should be referred to a FLS or an adequate, coordinated, multidisciplinary post-fracture prevention programme	1 to 2		9.50 (1.10)
				10 (6 to 10)
**7**	**Adherence to anti-osteoporosis medicines**			
	Non-physician HPs should address, monitor and support medication adherence in a structured follow-up.	2 to 3	B	8.83 (1.25)
				9 (6 to 10)

EULAR, European League Against Rheumatism; FLS, fracture liaison services; HPs, health professionals.

Box 1 **Research agenda to prevent and optimally manage fragility fractures for non-physician health professionals (HPs) including (but not limited to) dietitians, nurses, occupational therapists, pharmacists and physiotherapists**

- Randomised clinical trials on the effect of non-pharmacological interventions, as well as interventions to facilitate adherence.
- Research studies need to define and qualify those at high-risk of fragility fracture in patient sample populations.
- Research studies investigating interventions to prevent falls and fragility fractures need to clearly record fracture status at baseline.
- Validation and reliability testing of (multicomponent) screening methods for risk of falling is needed.
- Research studies need to include long-term follow-up measures of bone health, incidence rates of falls and fractures and functional mobility outcomes.
- A consensus agreement and statement between relevant stakeholders on the definition of high-risk of secondary fracture is required.
- Further clinical trials to evaluate the cost-effectiveness of management of patients with osteoporosis and/or a (high-risk) of fragility fractures by non-physician HPs are needed.
- Research studies to identify the clinically effective optimal duration, intensity and frequency of interventions delivered by non-physician HPs to patients following fragility fracture should be conducted.

Box 2 **Non-physician health professional (HP) education agenda to prevent and optimally manage fragility fractures**

**Non-physician HPs should be educated on:**

- How to use (multicomponent) screening tools to understand fracture risk.
- How to deliver, and what to include in a falls prevention programme.
- How to tailor education for people and patients with varying risk of falls.
- The scope and role of non-physician HPs in fracture liaison services.
- How to support and promote medication adherence.
- How to effectively promote bone health.
- Medication side effects that impact on bone health.

Education standards need to be agreed and underpinned by learning principles.

The task force met for one face-to-face meeting to review the results of the SLR and formulated the points to consider; these were finalised over subsequent weeks by online discussions and circulated to all task force members for voting via email. The level of agreement for the overarching principles and each point to consider was assessed using a numerical rating scale from 0 (complete disagreement) to 10 (complete agreement). In parallel with this, research and education agendas for the non-physician HP workforce to prevent and optimally manage fragility fractures were proposed and developed via a single round of iterative online discussion among the task force.

## Results

Two overarching principles to underpin high quality care were supported by the task force; shared decision-making^16^ and multi-professional working. Shared decision-making is an essential component of personalised care^17^ and may reduce unwarranted variation in healthcare practice,^18^ while involving non-physician HPs in the treatment and management of patients at high-risk of fragility fracture widens opportunities to prevent and optimally manage fragility fractures. Currently, non-physician HPs are only sometimes involved in the organisation and delivery of care for patients at high-risk of fracture.

Seven points to consider, describing non-pharmacological interventions, were developed and are summarised in table 2, along with underpinning levels of evidence, strength of recommendations and level of agreement among task force members.

### Point to consider 1: identification of patients at risk of fracture

No studies evaluating the effect of fracture risk detection by non-physician HPs were included in the SLR. Case finding people at risk of fracture can be undertaken in the first instance through identification of clinical factors (for example age, low body mass index, smoking, family fracture history, height loss ≥4 cm or a thoracic kyphosis).^19 20^ Simple online assessment tools incorporating various clinical risk factors (with or without a measure of BMD) into a fracture risk algorithm (such as the Fracture Risk Assessment Tool (FRAX),^21^ Garvan^22^ and QFracture^23^) are freely available in many countries^24^ and recent evidence suggests that FRAX-based screening and guided management of community-dwelling older women, may reduce incident hip fractures, but not overall fractures.^25^ Given the centrality of risk assessment to fracture prevention, the task force agreed that non-physician HPs should identify patients at risk of fragility fracture.

Risk identification and stratification can facilitate appropriate management, and workforce developments over recent decades have widened opportunities for non-physician HPs to manage individuals at risk of fragility fracture.^26 27^ National and local practice policies and pathways can be established to support requests for laboratory testing and diagnostic investigations (such as dual-energy X-ray absorptiometry scans) by non-physician HPs, and implementation of non-medical prescribing could increase patient access to effective osteoporosis treatment.^7 27^ As an example, Bowers *et al* 28 reported higher anti-fracture medicine prescription rates for women at high-risk of fragility fracture with implementation of a collaborative pharmacist-physician model of management compared with physician-only management.

### Point to consider 2: fall risk evaluation

Initial assessment of risk of falls in adults at high-risk of fragility fracture should focus on key questions relating to: any history of falls within the past 12 months, fear of falling and/or feeling unsteady while walking or standing.^29^ A positive response in any of these areas should be followed up with a multifactorial falls-risk assessment incorporating evaluation of gait and mobility (measured for example by the Timed Up and Go test^30^) and other relevant factors, such as balance, lower limb strength, medication, postural dizziness/hypotension, vision, mental health and cognitive capacity, footwear and environmental factors.^29^ Although the evidence identified in the SLR was insufficient to determine benefit of fall risk evaluation in adults at high-risk of fragility fracture, the task force agreed that a multifactorial falls risk assessment should be done (by one appropriately skilled HP or a number of different HPs^31^) as, when followed by multifactorial fall prevention interventions, multifactorial falls risk assessment involving non-physician HPs may reduce rate of falls in older people when compared with other approaches.^32 33^

### Point to consider 3: preventative multicomponent interventions

Multicomponent interventions, including for example exercise, fall-prevention strategies and education about bone health are important in primary fragility fracture prevention. Such multicomponent interventions may reduce fall rate and positively influence bone health in older people at high-risk of fragility fracture and/or at high-risk of falls.^34–36^

Regular long-term exercise is essential for bone health.^37 38^ Weight-bearing impact exercise and/or resistance training promotes strong bones and improves physical performance,^38^ while exercise interventions incorporating balance and functional training reduce rate of falls and number of fallers in older people at high-risk of falls living in the community.^39^

In people with bone fragility, we found sufficient evidence that multicomponent exercise incorporating dynamic weight-bearing, strength and balance training undertaken 2 to 3 days a week for at least 10 weeks, reduces risk of falling,^40^ and some evidence that multicomponent exercise undertaken for >1 year positively influences BMD.^35 41^ Evidence about whole body vibration or low impact exercise is limited and insufficient to determine effect on bone health-related outcomes in people with bone fragility.^42 43^

Customised multifactorial interventions, targeting individualised fall-risk factors, may reduce the incidence in falls rate in community-dwelling older people at high-risk of falling.^32 33^ One randomised controlled trial (RCT),^36^ reported a reduced falls rate in participants attending fall prevention clinics in Finland who received, on average, five fall and injury prevention interventions, commonly including home hazard modification, nutrition and lifestyle advice, medicines review and strength and balance training delivered by different HPs, including nurses and physiotherapists. The incidence rate of falls per 100 person years over a 12-month period were 95 in the intervention group and 131 in the control group (incidence rate ratio 0.72, 95% CI 0.61 to 0.86; p<0.001). The number needed to treat to prevent one fall was three.^36^

Data about the effect of nutrition on bone health-related outcomes in people with osteoporosis or osteopenia are limited. The evidence identified in our SLR was insufficient to determine the effect of vitamin D analogues, non-soy protein or daily vitamin K on BMD or fractures in older women with T scores between −1 and ≥−2.5.^44–46^ Nonetheless, maintenance of a healthy weight, increased consumption of fresh fruit and vegetables, lowering sodium intake and ensuring country-specific recommended intake levels of dietary calcium, may favourably impact bone health.^47^ Adequate serum levels of vitamin D are important for good musculoskeletal health, although the effect of supplementation on bone health-related outcomes remains contested.^48–50^ Analysis of pooled data from RCTs showed vitamin D supplementation had no effect on falls (n=34 144, relative risk (RR) 0.97, 95% CI 0.93 to 1.02) or total fractures (n=44 790, RR 1.00, 95% CI 0.93 to 1.07).^51^

The effect of face-to-face patient education on bone health-related outcomes in people with bone fragility is uncertain.^52^ In a systematic review including 13 RCTs of mostly high or moderate risk of bias, outcomes, including knowledge about osteoporosis, initiation and adherence to osteoporosis medication and fractures, were mixed;^52^ less than half of the studies reported a statistically significant difference favouring the intervention.

Despite insufficient evidence to determine the effect of some interventions, the task force agreed that non-physician HPs should offer multicomponent interventions including nutrition, multifactorial fall prevention initiatives and education, along with exercise (in particular supervised progressive weight-bearing, strength and balance training), to patients at high-risk of falls and or primary fragility fracture.

### Point to consider 4: avoidance of smoking and overuse of alcohol

The negative impact of tobacco smoking on bone and bone-health related outcomes are widely recognised.^53^ Smoking adversely affects bone mass in some populations,^54 55^ and results from meta-analyses consistently demonstrate increased risk of osteoporotic fractures in people who currently smoke compared with never or non-smokers.^56–59^

High intakes of alcohol (more than two units/day or ≥50 g/day) also increase fracture risk.^60 61^ The effects of alcohol on bone are complex and dose-dependent, and influenced by both direct and indirect mechanisms, such as alterations in activity and numbers of osteoblast and osteoclasts, hormonal changes and impaired nutrition.^62^ For some, the consequences of skeletal fragility are exacerbated by increased risk of falling^63^ mediated by intoxication and/or neuropathy.

### Point to consider 5: exercise and nutritional interventions for patients who have experienced a fragility fracture

Following hip fracture surgery, structured exercise interventions, in particular interventions that incorporate progressive resistance exercise training, result in small but significant improvements in mobility and physical function.^64 65^ Multicomponent exercise, incorporating strength and balance training, reduces risk of falls in people who have experienced an osteoporotic fracture,^40^ while regular long-term resistance and weight-bearing exercise may favourably affect BMD.^41^ Evidence about the optimal frequency, intensity and duration of exercise for people with osteoporotic fracture is limited. However, several country-specific recommendations drawing on expert consensus, in combination with evidence, are available to guide practice.^66 67^

Concerning the effect of nutrition on bone health, insufficient evidence was found to determine the effect of oral protein supplementation on functional outcomes in people following hip fracture^68^ while vitamin D (800 IU) and calcium (1000 mg) supplementation in older people with a history of osteoporotic fracture appeared generally ineffective in preventing future hip or any new fracture.^69^ One RCT, at low risk of bias, investigated the effect of a single loading dose of vitamin D3 compared with a placebo injection administered to older people within 7 days of hip fracture surgery.^70^ At 4 weeks there was no statistically significant between-group difference in fracture incidence, but the falls rate of participants in the active group was 250 (number of falls/days x 1000) compared with 821.4 in the placebo group (absolute risk reduction 57.1%).

The task force considered these findings and agreed that non-physician HPs should encourage adequate nutrition for patients with a history of osteoporotic fracture and discuss vitamin D and calcium intake with them, focussing on actual and recommended daily calcium intake, calcium and vitamin D rich foods and the individual’s risk/benefit profile for vitamin D supplementation.

### Point to consider 6: organisation and coordination of multidisciplinary services

The clinical and cost-effectiveness of coordinated multidisciplinary post-fracture models of care was confirmed in our SLR.^71–73^ Orthogeriatric services, delivering collaborative multidisciplinary inpatient care to older people admitted with hip fracture, reduce relative risk of in-hospital and long-term mortality compared with standard care. Functional recovery and factors associated with risk of falling may also be positively impacted by early multidisciplinary HP team care approaches.^74 75^

Alongside, multidisciplinary fracture liaison services (FLS), in which non-physician HPs such as nurses, pharmacists and physiotherapists effectively coordinate case finding, risk stratification and secondary fracture prevention,^76^ reduce re-fracture rates. In a meta-analysis of 19 519 participants who had experienced an osteoporotic fracture, a FLS compared with no FLS or usual care reduced absolute risk of re-fracture rate by approximately 30%.^72^ Irrespective of the care model or country, FLS when compared with usual care or no treatment are cost-effective.^73^

Many countries in Europe have now implemented coordinated post-fracture multidisciplinary models of care based on best practice standards,^77^ and the task force recommended that non-physician HPs should be included in these services.

### Point to consider 7: adherence to anti-osteoporosis medicines

Despite the efficacy of anti-fracture pharmaceuticals,^78 79^ rates of non-adherence to anti-osteoporosis medicines are high^80 81^ and adversely affect outcomes.^82^ Non-adherence to medicines can be characterised by non-initiation of a prescription, suboptimal implementation and premature discontinuation of treatment.^83^ Interventions to improve adherence commonly target drug regimens, systems, providers and patients, although effects are inconsistent in people with chronic health problems.^84^ There is some evidence that interventions delivered by HPs (education, less frequent dosing regimens, electronic prescription and pharmacist-delivered osteoporosis management services) may improve adherence to anti-osteoporosis medications.^85–87^ Consequently, the task force agreed that non-physician HPs should evaluate medication adherence in patients prescribed anti-osteoporosis medicines, and explore ways to improve adherence.

### Research and education agenda

The research and education agendas (boxes 1 and 2), support the development of capability and capacity within the non-physician workforce to prevent and optimally manage fragility fractures in adults 50 years or older. We recommend that consensus-derived core competencies are identified and embedded in HP education and training.

## Discussion

These EULAR points to consider, underpinned by shared decision-making and multi-professional working complement the previous EULAR/EFORT recommendations.^8^ They provide a template for the organisation and delivery of healthcare by non-physician HPs to prevent and manage fragility fractures and contribute to holistic patient management.^88^ In addition to fall risk evaluation and interventions delivered by non-physician HPs, the task force developed a separate point to consider, focussed on adherence to medicines. While some non-physician HPs prescribe medicines, all non-physician HPs should address, monitor and support adherence to prescribed anti-osteoporosis medicines in patients at risk of fragility fracture.

We acknowledge that patient management and HP roles and responsibilities differ across countries. However, these points can be tailored and used jointly by stakeholders as a focus for contextualised formative evaluations about implementation of interventions delivered by non-physician HPs, underpinned by country-specific patient level data from audit databases and registries.^89–91^ The generation of this knowledge, in conjunction with the identification of contextual barriers and facilitators to optimal management and implementation strategies,^92 93^ could enhance the role and impact of non-physician HPs working alongside medical colleagues to deliver services for this patient population.

We recommend that education about osteoporosis, fall and fracture risk assessment, and interventions to prevent and optimally manage fragility fractures, should be a core component of non-physician HP undergraduate training. An interdisciplinary focus through generic competencies for non-physician HPs in fragility fracture prevention and management, may lead to more consistent and effective care, and tackle the personal, societal and economic burden associated with fracture events.

The low levels of evidence for some points to consider call for well-designed research studies that include specific non-physician HP interventions. Such studies should consider using behavioural change techniques to enhance adherence to interventions delivered by non-physician HPs and optimise service delivery to prevent and manage fragility fractures.

Our study has some limitations. First, over half of our points to consider were formulated wholly or in part based on the expert opinion of the task force, due to insufficient published research evidence. Our definition of high-risk populations probably excluded evidence from other studies examining commonly used interventions, such as multifactorial falls prevention strategies for other older adult populations. Second, our SLR preferentially selected systematic reviews and large RCTs and may have excluded some studies. Third, while data extraction and risk of bias judgements were conducted systematically, duplicate independent assessments would have added further value. Lastly, the addition of a general practitioner on the task force would have been beneficial.

## Conclusion

The personal, societal and economic burdens associated with fragility fractures are enormous. These EULAR points to consider, based on robust development processes and agreed by an international task force, can guide non-physician HPs in the prevention and management of fragility fractures in adults 50 years or older.

10.1136/annrheumdis-2020-216931.supp2 Supplementary data
