# Supplementary material for: 2019 EULAR points to consider for non-physician health professionals to prevent and manage fragility fractures in adults 50 years or older
Source: Ann Rheum Dis. 2020 Apr 24;80(1):57–64. doi: 10.1136/annrheumdis-2020-216931 (PMC7788058; doi:10.1136/annrheumdis-2020-216931)
Supplement: Supplementary data [file annrheumdis-2020-216931supp001.pdf]

Supplementary File 1

Table 1. Clinical questions

1	Which diagnostic procedures, undertaken by non-physician health professionals, are recommended in the assessment of risk of falling in adults at high-risk of primary or secondary osteoporotic fracture?
2	What is the effect (including cost effectiveness and safety) of non-pharmacological treatments provided by non-physician health professionals after osteoporotic fracture?
3	What is the effect (including cost effectiveness and safety) of non-pharmacological treatments provided by health professionals in adults at high-risk of primary osteoporotic fracture?
4	What is the effect of strategies undertaken by health professionals to implement recommendations for the prevention and management of osteoporotic fracture by potential stakeholders?
5	What is the effect of multi-disciplinary team care on health outcomes for persons at high-risk of primary or secondary osteoporotic fracture?
6	What is the effect of interventions provided by health professionals to enhance adherence to anti-osteoporosis medicines in adults at high-risk of primary or secondary osteoporotic fracture?
7	What is the remit of the rheumatology review as undertaken by health professionals with respect to bone health across all rheumatic conditions?
8	What bone health education should health professionals deliver to people with rheumatic disease, specifically younger adults (up to 50 years of age)?

Supplementary File 1

Table 2: Characteristics of systematic reviews assessed against AMSTAR 2 criteria (Shea et al. 2017)

Author	PICO components	a priori protocol	Study design selection explained	Comprehensive search strategy	Duplicate selection	Duplicate extraction	Excluded studies listed	Included studies characteristics	Satisfactory ROB technique	Funding reported	Appropriate statistical methods in MA	Assessed impact of ROB on synthesis	Accounted for ROB in interpreting results	Explained heterogeneity satisfactorily	Adequately investigated publication bias	Reported conflict of interest
Avenell et al.	Y	Y	Y	Y	Y	Y	Y	Y	Y	N	Y	Y	Y	Y	Y	Y
Diong et al.	Y	Y	Y	Y	Y	Y	N	Y	Y	N	Y	Y	Y	Y	Y	Y
Lee et al.	Y	N	N	PY	N	N	N	PY	Y	N	Y	N	Y	Y	Y	Y
Liu et al.	Y	N	N	PY	N	N	N	PY	Y	N	Y	N	N	Y	N/A	Y
Newman et al.	Y	Y	Y	Y	Y	Y	Y	Y	Y	N	No MA conducted	No MA conducted	Y	Y	No MA conducted	Y
Visschedijk et al.	Y	N	N	PY	N	N	N	PY	N	N	No MA conducted	No MA conducted	Y	Y	No MA conducted	Y
De Kam et al.	Y	N	N	PY	N	N	N	PY	Y	N	No MA conducted	No MA conducted	N	Y	No MA conducted	Y
Luo et al.	Y	N	N	PY	N	Y	N	PY	Y	N	Y	Y	Y	Y	N/A	Y
Wei et al.	Y	Y	N	PY	Y	N	N	PY	Y	N	N	N	Y	N	N/A	Y
Varahra et al.	Y	Y	N	Y	Y	N	N	Y	Y	N	Y	Y	Y	Y	N/A	Y
Porter et al.	Y	Y	Y	PY	Y	Y	N	Y	Y	N	No MA conducted	No MA conducted	Y	Y	No MA conducted	Y
Koutsofta et al.	Y	N	N	PY	N	N	Y	Y	Y	N	No MA conducted	No MA conducted	Y	Y	No MA conducted	Y
Morfeld et al.	Y	N	Y	PY	Y	Y	Y	Y	Y	N	No MA conducted	No MA conducted	Y	Y	No MA conducted	Y
Grigoryan et al.	Y	N	N	PY	Y	N	N	N	Y	N	PY	N	N	Y	Y	Y
Wu et al.(a)	Y	N	Y	Y	Y	N	N	Y	Y	N	Y	Y	Y	Y	Y	Y
Wu et al.(b)	Y	N	Y	Y	Y	N	N	Y	Y	N	No MA conducted	No MA conducted	Y	Y	No MA conducted	Y
Hiligsman et al.	Y	N	N	PY	N	Y	Y	PY	Y	N	No MA conducted	No MA conducted	Y	Y	No MA conducted	Y

ROB, Risk of bias; MA, Meta-analysis; Y, Yes; N, No; PY, Partial Yes; N/A , Not applicable   Criteria not stated and or if no judgement could be made were annotated as No

Supplementary File 1

Table 3: Risk of bias assessment for the outcomes of interest: Randomised studies

	Sequence generation	Allocation concealment	Blinding of participants & personnel	Blinding of assessors	Incomplete outcome data	Selective reporting	Other bias
Mak et al.	Low	Low	Low	Low	Low*	Low	Low
Myint et al.	Unclear	Low	Unclear	Low*	Unclear	Low	Low
Kronborg et al.	Low	Unclear	Unclear	Low	Low	Low	Low
Mikó et al.	Low	Unclear	Unclear	Low	Low	Low	Low
De Morais Barbosa et al.	Unclear	Unclear	Unclear	High	Unclear	Low	Low
van Ooijen et al.	Unclear	Unclear	Unclear	Low	High	Low	Unclear
Di Monaco et al.	Low	Low	Unclear	Low	Unclear	Low	Low
Di Monaco et al.	Unclear	Unclear	Unclear	High	High	Low	Low
Berggren et al.	Unclear	Low	Unclear	High	Low	Low	Unclear
Shyu et al.	Low	Unclear	Unclear	High	Low	Low	Low
Cheung et al.	Low	Low	Low	Low	Low	Low	Low
Korpelainen et al.	Low	Unclear	Unclear	Low	Low	Low	Low
Gianoudis et al.	Low	Unclear	Unclear	Unclear	Low	Low	Low
Smulders et al.	Unclear	Unclear	Unclear	Low	Low	Low	Low
Palvanen et al.	Low	Low	Unclear	High	Low	Low	Low
Ciaschini et al.	Low	Unclear	Unclear	High	Low	Low	Low
Kilgore et al.	Unclear	Unclear	Unclear	Unclear	Unclear	Low	Unclear
Kennedy et al.	Low	Low	Unclear	Low	Low	Low	Unclear
Cox et al.	Low	Low	Unclear	Unclear	Low	Unclear	Unclear
Prestmo et al.	Low	Unclear	Unclear	Unclear	Low	Low	Low
Kooij et al.	Unclear	Unclear	Unclear	Low	Unclear	Low	Unclear

*For the outcome of interest

Table 4: Risk of bias assessment: Non randomised studies

	Recruitment & allocation concealment	Intervention integrity	Blinding	Incomplete outcome data	Selective outcome reporting	Other bias
Kemmler et al.	Unclear	Adequate (low)	Adequate (low)	Inadequate (high)	Adequate (low)	Unclear
Baypinar et al.	Unclear	Unclear	Unclear	Adequate (low)	Adequate (low)	Adequate (low)
Stuurman-Bieze et al.	Unclear	Unclear	Unclear	Adequate (low)	Adequate (low)	Unclear

Supplementary File 1

Table 5. Levels of Evidence modified from the Oxford Centre for Evidence-Based Medicine 2011 Levels of Evidence Table, OCEBM Levels of Evidence Working Group, The Oxford 2011 Levels of Evidence, Oxford Centre for Evidence-Based Medicine.
http://www.cebm.net/index.aspx?o=5653

Question	Level 1	Level 2	Level 3	Level 4	Level 5
**Does this intervention help?** (Treatment Benefits)	Systematic review of randomised trials or *n*-of-1 trials	Randomised trial or observation study with dramatic effect	Non-randomised controlled cohort/follow-up study	Case-series, case control, or historically controlled studies	Mechanism-based reasoning
**Is this (early detection) test worthwhile?** (Screening)	Systematic review of randomised trials	Randomised trial	Non-randomised controlled cohort/follow-up study	Case-series, case control, or historically controlled studies	Mechanism-based reasoning

Supplementary File 1

Table 6. Rating of available evidence (Modified from Ryan et al. 2014, p.11-12)

Summary statement	Translation
Sufficient evidence	• A statistically significant result from a large number of included studies/participants in a narrative synthesis or meta-analysis
Some evidence	• A more equivocal set of results from studies included in a narrative review, or a statistically significant result from meta-analysis of a small number of studies/participants and or low quality studies
Insufficient evidence	• Statistically non-significant results based on a small number of studies/participants potentially reflecting underpowering of the included studies OR<br>• Statistically non-significant results from a large number of studies/ participants reflecting underlying ineffectiveness on the outcome being examined and described as generally ineffective
Insufficient evidence to determine	• Where there are too few studies to be able to determine effect
